# Supplementary material for: Polarization-entangled photon pair sources based on spontaneous four wave mixing assisted by polarization mode dispersion
Source: Sci Rep. 2017 Jul 19;7:5785. doi: 10.1038/s41598-017-06010-8 (PMC5517469; doi:10.1038/s41598-017-06010-8)
Supplement: Supplementary file 1 — Supplementary Information [file 41598_2017_6010_MOESM1_ESM.pdf]

Supplementary Materials

Polarization-entangled photon pair sources based on spontaneous four wave mixing assisted by polarization mode dispersion

Pisek Kultavewuti[1,*], Eric Y. Zhu[1], Xing Xingxing[1], Li Qian[1], Vincenzo Pusino[2], Marc Sorel[2], J. Stewart Aitchison[1]

[1]Department of Electrical and Computer Engineering, University of Toronto, 10 King's College Road, Toronto, Ontario M5S 3G4, Canada

[2]School of Engineering, University of Glasgow, Glasgow G12 8QQ, Scotland, UK

Correspondence and requests for materials should be addressed to P.K. (email: pisek.kultavewuti@mail.utoronto.ca) or J.S.A (email: stewart.aitchison@utoronto.ca)

Biphoton wavefunctions

In Supplementary Figure 1, we show the magnitudes of the biphoton wavefunctions (BPFs) of all the eight SFWM processes (see equation (3) of the main article) in the 700-nm-wide and the 1,200-nm-wide waveguides, both with a waveguide length of 4.5 mm. The two waveguides represent the limiting cases of generating highly-polarization-entangled photon pairs: the 700-nm-wide waveguide is limited by the temporal walk-off (though not directly obvious in Supplementary Figure 1) whereas the 1,200-nm-wide waveguide is limited by state factorizability.

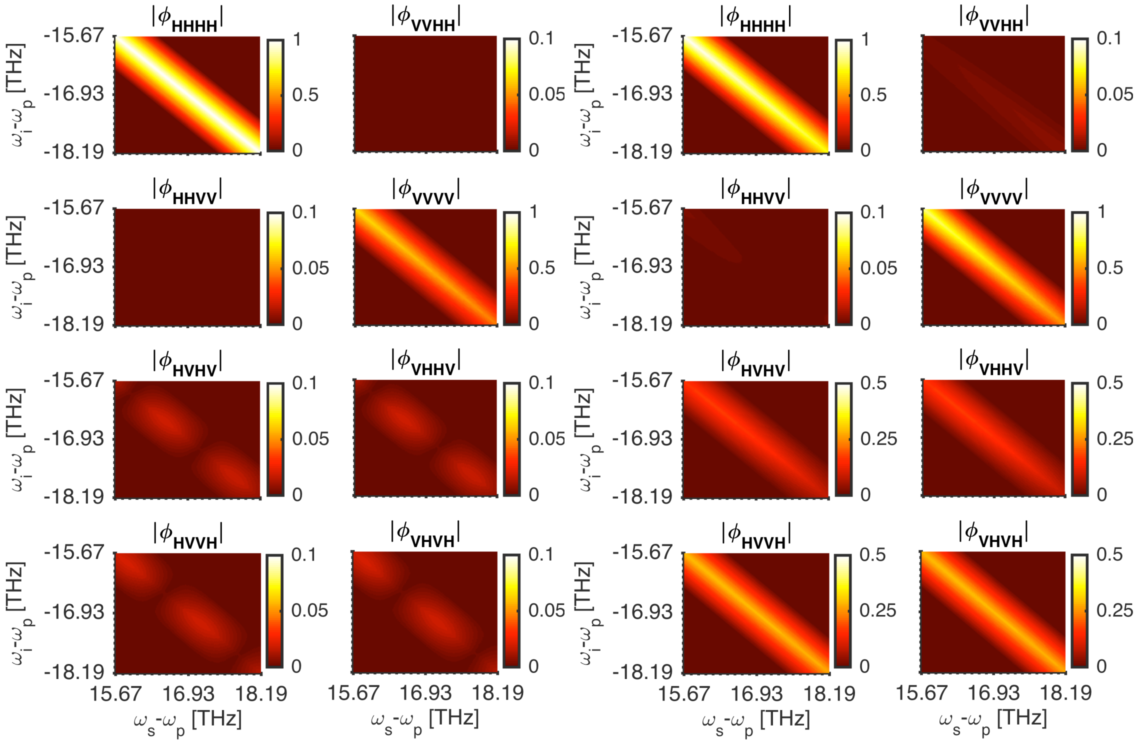

Supplementary Figure 1. Magnitudes of the biphoton wavefunctions of the eight SFWM processes as a result of orthogonally polarized pumps in (a) the 700-nm-wide waveguide and (b) the 1,200-nm-wide waveguide.

Supplementary Figure 2 displays the BPWs of the 1,100-nm-wide waveguide with the same waveguide length. This waveguide is the best performer, yielding the highest concurrence of 0.97±0.01, of the current experiment. Its dispersion property provides the optimal balance between the temporal walk-off and state factorizability, which is confirmed both in the calculation and experimentation.

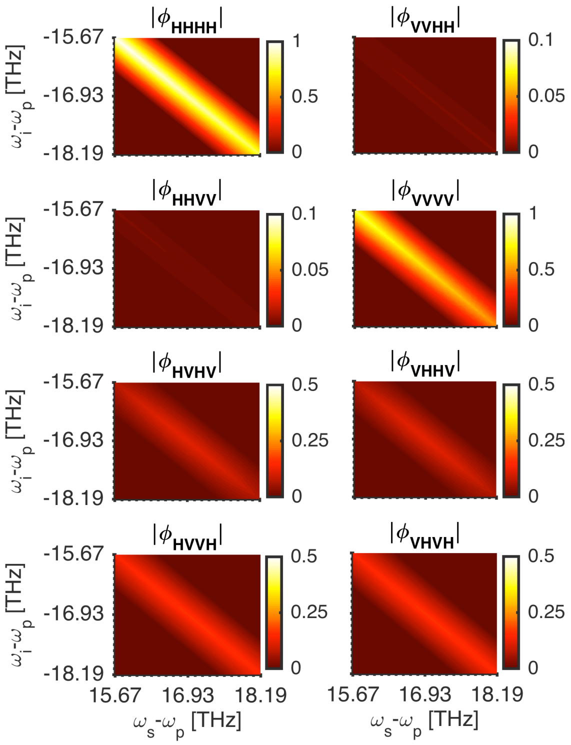

Supplementary Figure 2. Magnitudes of the biphoton wavefunctions of the eight SFWM processes as a result of orthogonally polarized pumps in the 1,100-nm-wide waveguide, which is the best performer in the current experiment.

State tomography measurement

Here, we show reconstructed density matrices from all the waveguides of the experiment. The density matrices are computed from the mean over a thousand instances of results from the maximum-likelihood state tomography with a Poisson randomization over the measured coincidence counts.

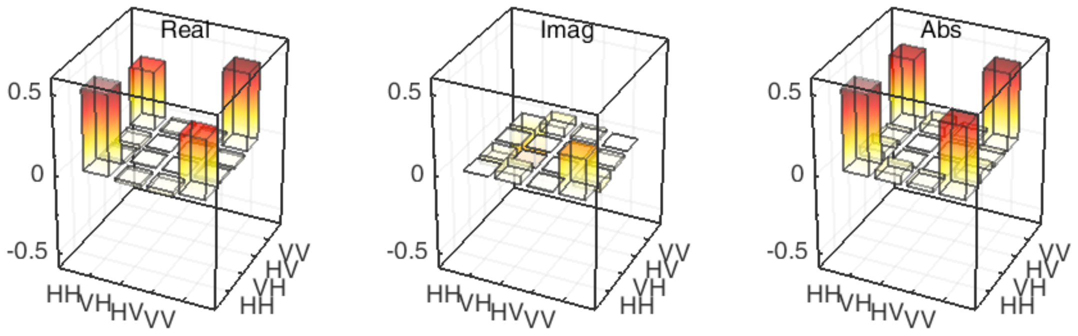

Supplementary Figure 3. Density matrix of the 700-nm-wide waveguide.

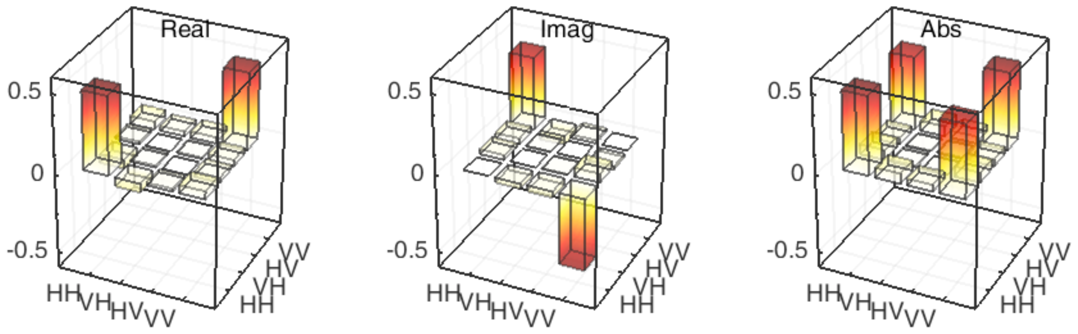

Supplementary Figure 4. Density matrix of the 750-nm-wide waveguide.

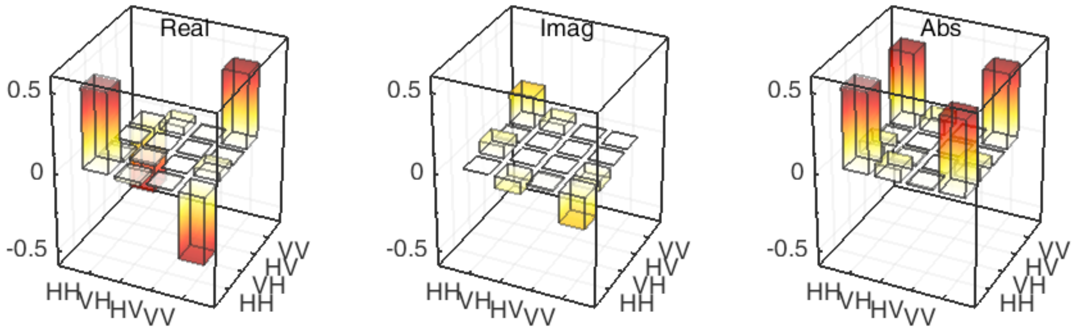

Supplementary Figure 5. Density matrix of the 800-nm-wide waveguide.

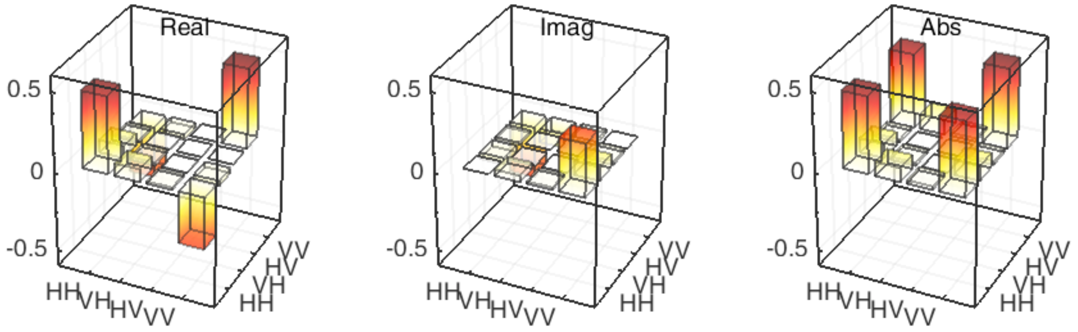

Supplementary Figure 6. Density matrix of the 900-nm-wide waveguide.

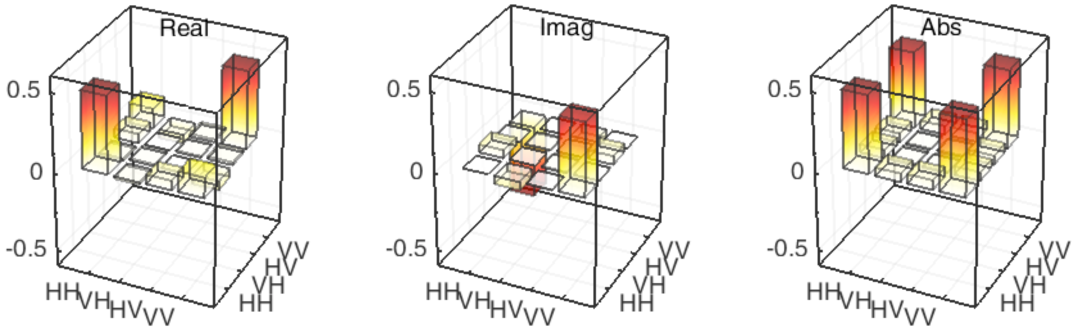

Supplementary Figure 7. Density matrix of the 950-nm-wide waveguide.

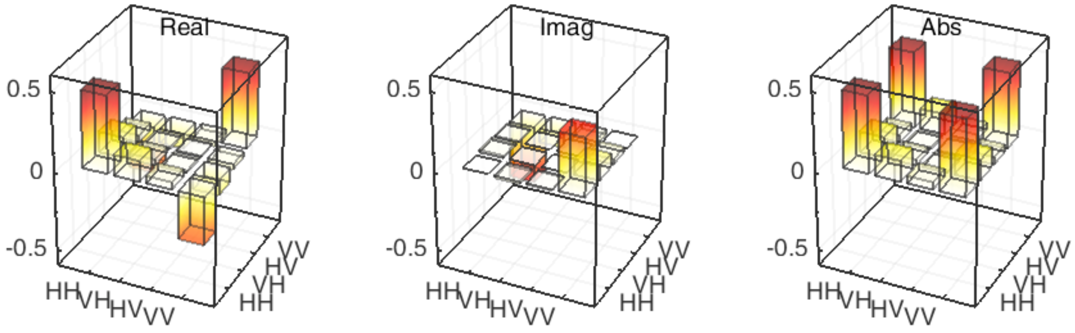

Supplementary Figure 8. Density matrix of the 1,100-nm-wide waveguide.

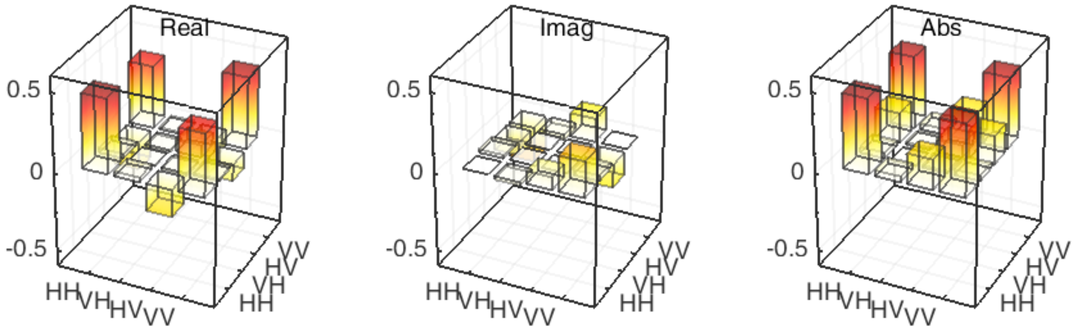

Supplementary Figure 9. Density matrix of the 1,200-nm-wide waveguide.

In addition, when projecting the state onto the $|HH\rangle$ or $|VV\rangle$ we could measure the coincidence-to-accidental ratio (CAR) of the co-polarized SFWM processes, i.e. HHHH=TE and VVVV=TM cases. The CAR of the TE and TM modes is shown in Supplementary Figure 10. The error is calculated from the measured coincidence and accidental counts according to the formula CAR = C/A. The CAR decreases in wider waveguides because the pump power was increased in order to maintain the photon pair generation rate in the quantum state tomography.

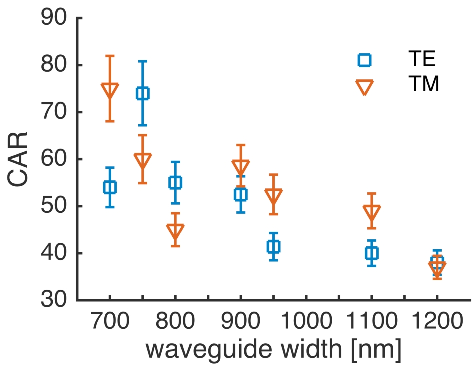

Supplementary Figure 10. Coincidence-to-accidental ratios of co-polarized SFWM processes (HHHH=TE and VVVV=TM) as a function of waveguide widths while the polarization-entangled state rate is maintained across the waveguides.

Predicting true coincidence and accidental counts

Given the theoretically predicted density matrix ρ and the state production rate μ, the true coincidence counts can be calculated from $C_t = \eta_s \eta_i \cdot \mu \mathrm{Tr}[|M\rangle\langle M|\rho]$ where $\eta_{s,i}$ is the total detection efficiency of the signal and idler channels and $|M\rangle\langle M|$ is the projection measurement. The accidental counts can be calculated from $A = s_s s_i$ where $s_{s,i}$ is the singles count of the signal and idler channels. The single counts are in turn calculated from $s = \eta(\mu \mathrm{Tr}[|M\rangle\langle M|\rho])(1+R)+d$ where d is the dark count rate and R captures of the ratio of the uncorrelated counts from spontaneous Raman scattering to the SFWM counts as studied[1]. The red curve in Fig. 5a of the main manuscript is calculated with $\mu = 0.016$, $\eta_s = -20$ dB, $\eta_i = -18$ dB, and $R = 0.74$.

Comparison of phase matching in $\chi^{(2)}$ and $\chi^{(3)}$ processes

In this section, we compare the phase matching condition, in particular the phase mismatch Δk, of the photon pair generation via the second-order $\chi^{(2)}$ process and the third-order $\chi^{(3)}$ process in a AlGaAs waveguide with a geometry similar to our waveguides in this study. For both processes, the power of the down-converted photon is proportional[2] to $\mathrm{sinc}^2(\Delta kL/2)$, and it drops to a half when $\Delta kL/2 = 1.39$. The phase mismatch of the $\chi^{(2)}$ process can be estimated using a reported second harmonic generation in Duchesne *et al*[3]. Using the same waveguide length L of 5 mm as in ours, the phase mismatch of the $\chi^{(2)}$ process increases from 0 to 556 m^{-1} when the pump wavelength is detuned from its perfect phase matching condition by about 0.5 nm. For the $\chi^{(3)}$ process in our work, we apply the formula $\Delta k = k^{(2)}(\Delta\omega)^2/2$ and use $k^{(2)} = 1\ \mathrm{ps^2m^{-1}}$ and $\Delta\omega = 1$ Trad (~1 nm in wavelength detuning), then we get $\Delta k = 0.5\ \mathrm{m^{-1}}$, compared to 556 m^{-1} of the $\chi^{(2)}$ process. Since $k^{(2)} = 1\ \mathrm{ps^2m^{-1}}$ or lower over a wide range of pump wavelengths (see Figure 1 of the main paper), the $\chi^{(3)}$ process therefore has a ready phase matching. Moreover, the down converted signal and idler photon pairs can be generated over broad frequency conjugates. For instance, the photon pair can be generated over a 60-nm bandwidth[1] centering at the pump wavelength given the $k^{(2)} = 1\ \mathrm{ps^2m^{-1}}$.

References

1. Kultavewuti, P. *et al.* Correlated photon pair generation in AlGaAs nanowaveguides via spontaneous four-wave mixing. *Opt. Express* **24,** 3365–3376 (2016).
2. Helt, L. G., Liscidini, M. & Sipe, J. E. How does it scale? Comparing quantum and classical nonlinear optical processes in integrated devices. *J. Opt. Soc. Am. B* **29,** 2199 (2012).
3. Duchesne, D. *et al.* Second harmonic generation in AlGaAs photonic wires using low power continuous wave light. *Opt. Express* **19,** 12408–17 (2011).
